# Foreword

**DOI:** 10.1155/EDR.2003.203

**Published:** 2003

**Authors:** Michael Brownlee


*Experimental Diab. Res.*, 4:203–204, 2003
Copyright © Taylor and Francis Inc.
ISSN: 1543-8600 print / 1543-8619 online
DOI: 10.1080/15438600390269301

Taylor & Francis
Taylor & Francis Group

### Foreword

Chronic hyperglycemia and development of diabetes-specific microvascular complications in the retina, renal glomerulus, and peripheral nerve are characteristic of all forms of diabetes. As a consequence of its microvascular pathology, diabetes is the leading cause of blindness, end-stage renal disease, and a variety of debilitating neuropathies. Diabetics are the fastest growing group of renal dialysis and transplant recipients, and in the United States, their 5-year survival rate is only 21%, worse overall than that for all forms of cancer combined. Over 60% of diabetic patients suffer from neuropathy, which includes mononeuropathies, distal symmetrical polyneuropathy, and a variety of autonomic neuropathies causing erectile dysfunction, urinary incontinence, gastroparesis, and nocturnal diarrhea. Fifty percent of all non-traumatic amputations in the United States are due to diabetic peripheral neuropathy, often in conjunction with lower extremity arterial disease.

The causal link between diabetes and microvascular complications is chronic hyperglycemia. Large prospective clinical studies in both Type I and Type II diabetics have demonstrated a strong relationship between glycemia and diabetic microvascular complications.

Diabetes-specific microvascular disease in the retina, glomerulus, and peripheral nerve is characterized by shared pathophysiologic features. A large body of evidence from clinical trials and animal studies has demonstrated that chronic hyperglycemia is the critical initiating factor for all types of microvascular disease. Both duration and magnitude of hyperglycemia are strongly correlated with the extent and rate of progression of diabetic microvascular complications. Although all diabetic cells are exposed to elevated levels of plasma glucose, hyperglycemic damage is limited to those cell types (e.g. endothelial cells) that are unable to downregulate glucose transport into the cell, leading to intracellular hyperglycemia. Further evidence that intracellular hyperglycemia is the critical factor in the development of diabetic pathology is the fact that GLUT-1 overexpression in mesangial cells cultured under normoglycemic conditions induces the same increase of collagen type I and IV, and fibronectin gene expression as does hyperglycemia in mesangial cells that don't overexpress GLUT-1.

Early in the course of diabetes, in the absence of structural changes, intracellular hyperglycemia causes abnormalities in blood flow and increased vascular permeability. The resulting increase in blood flow and intracapillary pressure reflects decreased activity of vasodilators such as nitric oxide, increased activity of vasoconstrictors such as angiotensin II and endothelin-1, and elaboration of permeability factors such as VEGF. As a consequence, retinal capillaries exhibit increased leakage of flourescein and glomerular capillaries show an increased albumin excretion rate. Initially this process is reversible, however quantitative and qualitative abnormalities of the extracellular matrix contribute to an irreversible increase in vascular permeability. With time, microvascular cell loss, in part due to programmed cell death, and progressive capillary occlusion occur, due both to extracellular matrix overproduction induced by growth factors such as TGF$\beta$, and to deposition of extravasated periodic acid-Schiff-positive plasma proteins. Hyperglycemia may also decrease production of trophic factors for endothelial and neuronal cells. Connective tissue growth factor (CTGF), a key intermediate molecule involved in the pathogenesis of fibrosing chronic diseases, has recently been shown to be overexpressed in kidney, myocardium and aorta from diabetic animals as well, implicating CTGF in the pathogenesis of both micro- and macrovascular diabetic complications. Together, the pathophysiologic events described above lead to edema, ischemia, and hypoxia-induced neovascularization in the retina, to proteinuria, mesangial matrix expansion and glomerulosclerosis in the kidney, and to multifocal axonal degeneration in peripheral nerves.

In this timely and important special issue, the authors add valuable new information, about an emerging paradigm in the pathogenesis of diabetic complications: the role of decreased levels of critical trophic factors such as insulin like growth factor I (IGF-1), nerve growth factor (NGF) and related neuropeptides, and proinsulin-derived C-peptide. This paradigm has obvious immediate therapeutic implications, because replacing abnormally low levels of trophic factors may well have a beneficial effect in the prevention and treatment of diabetic complications.

Like all good science, this special issue of *Experimental Diabesity Research* raises new important questions as well. First, it is now well-established that each of the four major biochemical pathways underlying diabetic complications reflect a single hyperglycemia-induced process: overproduction of superoxide by the mitochondrial electron transport chain. How does this process lead to changes in expression of trophic factors, damaging cytokines and growth factors, and their receptors? Even more fascinating is the difference in IGF-1 expression in Type I vs Type II diabetic patients. These data raise the question, now being asked under the rubric of "Systems Biology," about the importance of the temporal, and well as the quantitative dimension. For both hyperlglycemic exposure, as well as cytokine and growth factor exposure, the temporal pattern, both short- and long-term, are likely to play a very important role in determining the net effect of both. After reading this publication, I am more convinced than ever that the answers to these important questions will emerge over the next few years.

*Michael Brownlee, M.D.*
*New York, August, 2003*

